# Analysis of qPCR Data: From PCR Efficiency to Absolute Target Quantity

**DOI:** 10.3390/ijms262411885

**Published:** 2025-12-09

**Authors:** Jan M. Ruijter, Maurice J. B. van den Hoff

**Affiliations:** Department of Medical Biology, Amsterdam UMC, Location AMC, Meibergdreef 15, 1105AZ Amsterdam, The Netherlands

**Keywords:** qPCR, PCR efficiency, amplification curves, absolute quantification, baseline correction, quantification threshold, reaction components, limiting conditions

## Abstract

Quantitative Polymerase Chain Reaction (qPCR) is a very sensitive method to determine small amounts of DNA or RNA in experimental, environmental, veterinary, forensic and clinical samples. Despite efforts from the qPCR community to address qPCR variability by recommending standardization of reporting of all steps of a qPCR experiment, most reported qPCR results are still grossly biased. The first part of this paper describes two decades of efforts to remedy this situation by promoting so-called efficiency-corrected qPCR data analysis. Although such analysis leads to less variable qPCR results, the outcome, fluorescence at cycle zero, is difficult to grasp. In the second part, we outline how qPCR analyses can result in N_copy_, the number of copies of the target at the start of the reaction. A newly developed theoretical approach determines N_copy_ using the characteristics of the amplification curve and the known concentrations of all reaction components. By including these reaction-mix characteristics in the analysis, this N_copy_ is assay-, machine- and laboratory-independent and thus allows direct worldwide comparisons. Moreover, N_copy_ provides a very intuitive and easy-to-interpret absolute quantitative result.

## 1. Introduction

Since its inception in the early nineties of the last century [1,2], quantitative Polymerase Chain Reaction (qPCR) has evolved into the worldwide method of choice to detect small amounts of DNA or RNA reflecting the presence of pathogens or organisms in samples collected in clinical, environmental and forensic research or measure the expression level of genes in samples collected in basic and experimental research [3,4]. qPCR is considered to be a very sensitive and precise method to determine the number of copies of a target of interest in a given sample. However, reproducibility of qPCR results has been the subject of discussion for decades [5,6,7]. Despite the efforts to standardize the reporting of all steps of a qPCR experiment [4,8,9], crucial steps in the analysis of qPCR data are still widely ignored, and therefore grossly biased qPCR results are reported and accepted by reviewers and journals [10,11,12]. The first part of this paper describes our two decades of efforts to remedy part of this situation by promoting so-called efficiency-corrected qPCR data analysis [13,14,15]. In the second part, we describe several qPCR analysis methods that allow the actual number of target copies at the start of a reaction (N_copy_) to be calculated and outline a newly developed approach using the characteristics of the amplification curve and the knowledge about the concentrations of reaction components to determine that number of target copies.

## 2. The qPCR Basics

### 2.1. PCR Kinetics

Each reaction in a PCR run comprises the sample to be measured, a heat-stable DNA polymerase, a reaction buffer, nucleotides, primers and a fluorescent DNA-binding dye or probe [16]. The forward and reverse primer pair is designed such that it specifically flanks the so-called amplicon: the target of interest (toi) region in the DNA. During a qPCR run the reaction mixture is repeatedly heated and cooled in so-called amplification cycles. One amplification cycle comprises heating the reaction to 95 °C to denature the DNA into single strands, cooling to the annealing temperature, which is chosen such that the primers specifically and uniquely bind the targeted sequence, followed by heating to 72 °C at which the heat-stable DNA polymerase synthesizes the complementary DNA strands starting at the forward or reverse primer resulting in two double strand amplicons. In clinical studies, a two-step PCR reaction is often performed, in which both the annealing of the primers and subsequent DNA synthesis are executed at the same temperature [17]. In both setups, the exponential accumulation of the amplicon during the PCR run is described by the kinetic equation for exponential growth N_C_ = N_0_E^C^ [14]. In words, the number of amplicons after a given amplification cycle (N_C_) is equal to the number of copies of the target at the start of the PCR (N_0_) times the efficiency of the amplification reaction (E) to the power the given cycle (C). The amplification efficiency is thus defined as the fold-increase per cycle and has a value between 1 and 2, where 2 is a 100% efficient reaction in which in each cycle the number of amplicons is doubled. In this paper the symbol N_0_ will also be referred to as the target quantity.

### 2.2. Fluorescence Monitoring During the PCR

**Figure 1:**
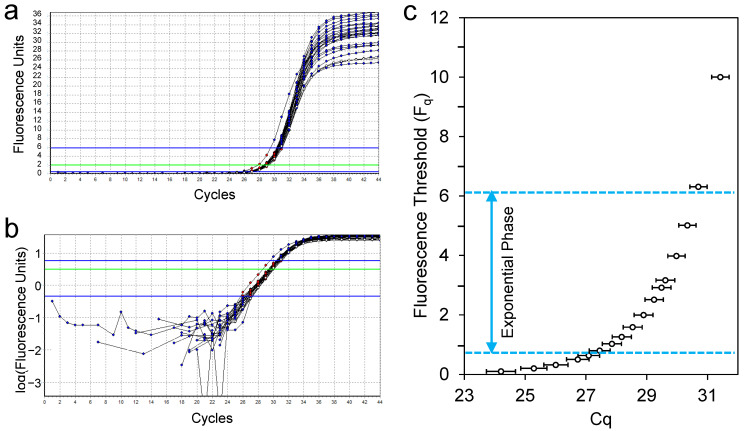
Amplification curves and quantification threshold setting. (**a**) Amplification curves of a set of 24 positive control reactions from a COVID assay run on the Lightcycler 480 II and analyzed with LinRegPCR [14,15] plotted on a linear fluorescence scale. (**b**) The same amplification curves plotted on a logarithmic fluorescence scale. The straight exponential phase is found between the blue lines. The green line in panels (**a**,**b**) is an example of a set fluorescence threshold (F_q_). (**c**) Range of Cq values (mean and standard deviation) obtained after setting the quantification threshold (F_q_) from low to high.

Because it is impossible to directly measure the number of target molecules at the start of the PCR, the number of amplicons at every cycle of a reaction is monitored indirectly by measuring the fluorescence of a dye or probe [18,19]. As a consequence, a qPCR machine reports fluorescence values (F) that are corrected for system background fluorescence, which is the fluorescence that is independent of the monitoring chemistry and cycling [20]. The kinetic equation of PCR should actually be written as F_C_ = F_0_E^C^. These fluorescence values can be used to plot the amplification curve. In everyday practice, the exponential phase of a PCR is only a limited number of cycles in the amplification curve (Figure 1a,b). This exponential phase is preceded by a ground phase, in which the amplicon is exponentially amplified but the fluorescence resulting from monitoring these amplicons is insufficient to exceed the baseline fluorescence. This baseline fluorescence is defined as the fluorescence of the PCR monitoring dye or probe but is independent of the amplification. The exponential phase ends when one of the reaction components becomes limiting, and consequently the observed PCR efficiency starts to decrease. This decrease in PCR efficiency is reflected in the amplification curve as the transition phase towards the plateau phase. In the latter phase the fluorescence remains constant.

### 2.3. Common Analysis of qPCR Data

Analysis of qPCR data commonly consists of the following basic steps: (1) subtraction of baseline fluorescence, (2) setting of the quantification threshold (referred to as F_q_) and determining how many cycles were needed to reach that fluorescence threshold, a value referred to as the fractional quantification cycle (Cq) [19] and (3) determining the PCR efficiency (E) [21]. The inverse of the kinetic equation of PCR, F_0_ = F_q_/E^Cq^, can then be used to calculate F_0_, the fluorescence associated with the target quantity [13]. Early in the qPCR era, researchers preferred to report the expression level of a gene of interest relative to a reference gene (often still erroneously referred to as housekeeping gene) or even as the fold difference of the relative expression level between treated and control conditions. Simple mathematics with the assumption that the PCR efficiency is always 100% then showed that F_q_ and E were cancelled out from the above calculation, and qPCR results could be reported as Cq values or differences between Cq values [22]. The bias introduced by this practice has been the subject of our papers [11,15]. For now, it suffices to say that because of more than two decades of these Cq-based results being reported, the crucial role of the PCR efficiency in qPCR data analysis has been forgotten by the majority of researchers. To illustrate the importance of these basic steps in qPCR analysis, we will discuss them separately in Section 2.3.1, Section 2.3.2, Section 2.3.3.

#### 2.3.1. Removing Baseline Fluorescence

**Figure 2:**
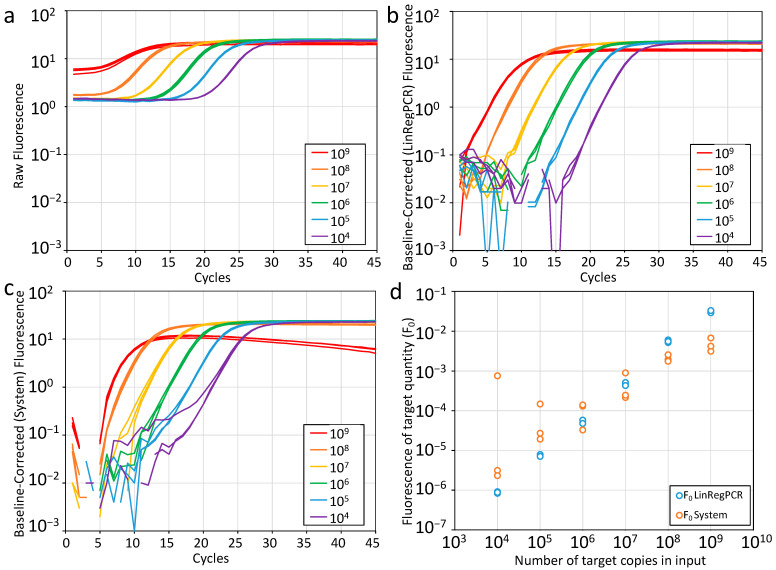
Comparison of baseline correction by LinRegPCR [14] and the qPCR system. The amplification data in this figure are from a dilution series starting with 10^9^ copies of a double-strand plasmid encoding human cardiac troponin (TNNT) used for validation of an assay and amplified on the LightCycler 480 II using the Roche SYBR Green I master mix. (**a**) Raw fluorescence data exported by the qPCR system. Colors indicate the number of target copies in the triplicate reactions per concentration. (**b**) Amplification curves after baseline correction with LinRegPCR. (**c**) Amplification curves after system baseline correction with a trendline fitted to the data points in cycles 2–5. (**d**) Relation between the fluorescence of the target quantity (F_0_, y-axis) and the number of targets in the input (x-axis) determined after baseline correction by LinRegPCR (blue) or the qPCR system (orange).

Although the software of the PCR machine removes the system’s background fluorescence, the measured fluorescence values still contain the monitoring chemistry-dependent, but amplification-independent, baseline fluorescence (Figure 2a). For correct analysis of the amplification curve, this baseline fluorescence also needs to be removed. Various methods to subtract this baseline fluorescence have been implemented in qPCR machine software. The original method was to calculate the mean fluorescence of a user-defined number of ground phase cycles. Subsequently, this mean value was subtracted as the baseline value from the measured fluorescence of each amplification cycle. However, early in this millennium all qPCR machines switched to fitting a straight trendline through the fluorescence values observed in a machine- or user-defined number of early cycles and subtracting the calculated values of this trendline from the fluorescence value observed in each of the amplification cycles [20] (Figure 2c).

Although an influential paper in qPCR analysis mentioned the need to adjust the ground phase cycles when the amplification curves showed a deviating shape [23], not all qPCR users may have been aware of their responsibility to set the correct number of ground phase cycles when evaluating their qPCR data. Moreover, estimating a baseline value from the early PCR cycles suffers from some major issues. The first cycles of a PCR have very low fluorescence values, which show considerable variation, if not the most variation of the entire run. This is especially true when the sensitivity of the photomultiplier is set to dynamic. It is advised to disable this setting and always measure fluorescence in a fixed time window, e.g., 0.25 s. However, even then the variation in ground phase fluorescence values still affects the baseline, resulting in an increasing or decreasing trend which is propagated linearly into the fluorescence values of the baseline-corrected amplification curve (Figure 2c,d). Another issue of determining the baseline from the early cycles is that for reactions with a (very) high target input, the fluorescence values of the first cycles of the exponential phase may become included in some, if not all, cycles that are included in the ground phase. For such reactions, the baseline trend line may become (very) steep, which will even lead to decreasing plateau values. In extreme cases, when the target quantity is less than two orders of magnitude below the number of primer molecules in the reaction, the fluorescence observed in the first cycles may already be close to the plateau level. In that case, subtracting the ground phase-based baseline will result in an amplification curve that does not reach the quantification threshold. As a consequence, this would lead to the erroneous conclusion that in such a reaction, e.g., a clinical sample, the target is not present at all. Overall, our conclusion was and still is that the early cycles of a PCR should not be used to estimate the baseline fluorescence [14]. How to estimate the baseline fluorescence without using early cycles will be discussed in Section 2.4.1.

#### 2.3.2. Setting the Quantification Threshold

In the classic qPCR analysis, a quantification threshold (F_q_) is set by the qPCR machine or the user. The intersection of the baseline-corrected amplification curve with this threshold is determined and used to calculate the quantification cycle, which is the fractional number of cycles that were needed to reach the threshold. This value is referred to as Cq and is generally considered to be a measure of the amount of the target of interest at the start of the reaction [8]. Recommendations for the setting of this quantification threshold changed over time from low, to avoid differences in the start of the transition into the plateau phase, to high, to avoid noise in the ground phase cycles [19]. What these recommendations have in common is that they state that the threshold should be set in the exponential phase of the reaction. However, it should be noted that this exponential phase can only be identified unequivocally when the amplification curve is plotted with a logarithmic fluorescence axis, which is not the default setting in most qPCR machines (Figure 1a,b).

At this point it is very important to realize that different threshold levels will give different Cq values (Figure 1c). Worryingly, many qPCR papers report Cq values, differences in Cq values (ΔCq) and differences of those differences (ΔΔCq) as the primary outcomes of a qPCR-based experiment [24]. Standardization of threshold setting within and between experiments is crucial when these Cq values are compared or reported as the only outcome of an experiment. In that case, the quantification threshold should be set at the same level for all reactions, amplicons and runs to allow these calculations and valid comparison and interpretation of these results [11]. Some qPCR machines automatically set a threshold per run based on the observed fluorescence values, thus hampering any comparison of Cq values between runs and, therefore, experiments, papers and laboratories [12]. For the same reasons the setting of a Cq cut-off in a clinical assay to identify positive reactions is not recommended [25].

In conclusion, to avoid the biases introduced by the threshold setting when Cq values are reported, this threshold setting has to be standardized between targets, within laboratories and between laboratories (Figure 1c). However, in anticipation of our proposed solution, a far easier way to accomplish standardization is to abandon the erroneous practice of reporting qPCR results as Cq, ΔCq or ΔΔCq values completely and report qPCR results as F_0_ or, preferably, N_0_ [15].

#### 2.3.3. Determining the PCR Efficiency

As early as 2001, the bias introduced by reporting ΔCq values and thus assuming a PCR efficiency of 100% was considered incorrect and to be avoided. Still adhering to the difference in Cq values approach, efficiency-corrected relative quantification was introduced [26]. In this approach, differences in Cq values were combined with the PCR efficiency of the assay for each target. This efficiency is generally calculated from the slope of a standard curve generated with using a series of dilutions of a standard sample containing the target of interest [19,27,28,29]. When no such standard is available, one of the positive samples can be used because it is only the dilution that is used in the calculation of the PCR efficiency. Preferably, the biological matrix complexity of the diluted standard should be similar to that of the unknown samples that are to be analyzed. The Cq values of the standard curve sample (y-axis) are then plotted versus the logarithm of the dilution of the standards (x-axis). The slope of the linear regression line fitted to these data can be used to calculate the PCR efficiency. In this paper, this PCR efficiency is referred to as the standard curve (here abbreviated to stc)-derived PCR efficiency and is calculated with E_stc_ = 10^(−1/slope)^ [20,30]. To obtain a reliable efficiency value, the standard curve’s concentration range should cover 4–5 orders of magnitude and include at least three technical replicates for each dilution. Validity of the standard curve can be judged from its slope, intercept, residual variation, LoD, LoQ and r^2^ value, which should be reported for each assay [4].

A major issue with standard curves is that the x-axis represents the logarithm of the intended dilution. The actual dilution may, however, be different because of a pipette calibration error, which will be propagated during the serial dilution preparing the standard curve. Such systematic errors in the serial dilution will go unnoticed but will have relatively large consequences for the standard curve slope and thus for the calculated PCR efficiency [13,14,15]. When different pipettes are used for dispensing the DNA sample and the buffer, both have to be correctly calibrated [31]. It is also important to note that the presence of a PCR inhibitor or stimulator in the stock solution used to prepare the standard curve will go unnoticed [13], but its dilution will also affect the slope of the regression line and thus the derived PCR efficiency [25,32]. A further caveat of this approach is that such a standard curve has to be prepared for each PCR run due to between-run differences [10,33,34,35,36].

Taken together, determining the PCR efficiency from dilution series is very labor-intensive and costly and often does not provide the intended accuracy. Therefore, we do not recommend using this approach. Nevertheless, the PCR efficiency has to be determined because the efficiency-corrected approach of qPCR data analysis is the current accepted method, as recommended in the MIQE 2.0 guidelines [4]. Fortunately, correct PCR efficiency can be determined without dilution series as will be described in Section 2.4.3.

#### 2.3.4. Comparison of qPCR Data Analysis Methods

The above three paragraphs illustrate that the commonly used way to perform each of the three basic steps in qPCR analysis leads to errors and bias in the reported results. In the first decade of this millennium, the growing awareness of these problems has led to the development, publication and implementation of a number of PCR data analysis methods [29,37,38,39,40,41,42,43].

To help users choose between these methods, in 2013 their developers made the combined effort to compare the performance of these methods by each analyzing the same large set of qPCR data with their programs and comparing their outcomes [21]. To our knowledge, no new amplification curve analysis approaches have been published since then. This comparison of amplification curve analysis methods showed that the best performance was reached when a PCR efficiency per assay was used, rather than methods that used a PCR efficiency per reaction. The latter methods showed more variable and less reproducible results. The methods that derived the PCR efficiency from standard curves showed the least bias because the results were compared to the input calculated with the intended dilution. When the PCR efficiency was determined from the amplification curves and averaged per assay, the calculated target quantities were more reproducible. But because this result of the standard curve was compared with the intended input, not the actual input, it was concluded that this method showed the most bias. However, because calculation of the PCR efficiency from a standard curve uses the intended rather than the actual dilution, the low bias of the standard curve approach is the result of circular reasoning [15,21]. Excluding this performance indicator from the analysis of the different methods [21], the LinRegPCR program showed the best performance. In Section 2.4 we will describe how LinRegPCR handles the basic steps of qPCR data analysis to obtain this superior outcome.

### 2.4. LinRegPCR Approach to qPCR Data Analysis

#### 2.4.1. Baseline Subtraction Revisited

Amplification curve analysis with LinRegPCR starts with importing raw, i.e., background but not baseline corrected, fluorescence data, from the qPCR machine [14,44]. When the fluorescence baseline is then subtracted correctly, the exponential phase of the amplification curve will reflect the kinetic equation of PCR. This means that plotted on a logarithmic fluorescence axis, this exponential phase is a straight line (Figure 1b and Figure 2b). If the subtracted baseline value is too high, the amplification curve will bend downward, and early cycles will be lost. When the subtracted baseline value is too low, the amplification curve will bend upwards and may even show a flat early phase [20]. LinRegPCR utilizes this effect of baseline correction on the linearity of the exponential phase of the amplification curve. It uses an iterative approach to determine the baseline value that results in the longest straight line through the exponential phase [14]. This constant baseline value is then subtracted from all observed fluorescence values in that reaction. Note that this baseline estimation has to be performed for each individual amplification curve. The main advantage of this approach is that the ground phase values are not used, which means that the large variation in the ground phase fluorescence values is not propagated in the further analysis of the qPCR data. Moreover, the other issues that were described to occur with high or extreme target quantities are avoided (Figure 2b,c).

#### 2.4.2. Quantification Threshold Setting

As discussed above, valid qPCR data analysis requires that the setting of the quantification threshold is standardized per run. To this end, the LinRegPCR program sets the quantification threshold at the end of the exponential phase. The latter is marked in each amplification curve as the cycle where the fold-increase per cycle starts to decrease. This point is generally referred to as the second derivative maximum (SDM). Because this SDM can vary between targets and differs between reactions, LinRegPCR calculates the mean of the fluorescence values at the SDM per run and sets the quantification threshold (F_q_) one cycle below this mean fluorescence for the entire run. Note that because of our decision to calculate and publish F_0_ results per reaction, standardization of the threshold per target should suffice. The setting of the threshold per run is implemented only to accommodate users who use the LinRegPCR program to obtain the reported Cq values per reaction. It should, however, be noted that this common threshold per run does not allow for inter-run comparison of these Cq values.

#### 2.4.3. PCR Efficiency Determined from Amplification Curves

The kinetic equation of PCR shows that the slope of the line through the data points in the exponential phase of the baseline-corrected amplification curve is determined by the PCR efficiency [13,14]. Using this property of the amplification curve (here abbreviated to amc), the program determines the PCR efficiency per reaction with E_amc_ = 10^slope^. It should be taken into account that in the baseline-corrected fluorescence values, the fluorescence values in early exponential phase cycles may still be affected by baseline noise, whereas fluorescence values in late exponential phase cycles may be affected by variation in the start of the transition phase. To avoid both of these issues, LinRegPCR sets a so-called window of linearity. In an iterative algorithm, it searches for the fluorescence window containing four cycles for each reaction in which the coefficient of variation of the PCR efficiency values is lowest (Figure 1b). Because the PCR efficiency differs between assays, this window of linearity is set per target [14,44]. For each target within the run, the mean E_amc_ is then calculated and used to calculate the F_0_ per reaction (F_0_ = F_C_/E_amc_^Cq^). In a clinical or veterinary setting and with point-of-care qPCR assays in which the specimen sample is directly added to the reaction mixture without purification, better results may be obtained when a specimen-specific, rather than a target-specific, E_amc_ is used. In this specific case, replicate reactions per specimen will improve the results because then a specimen-specific mean E_amc_ can be used [15].

#### 2.4.4. Reporting Results with F_0_ Values

As described above, the inverse of the kinetic equation of PCR (F_C_ = F_0_ E_amc_^Cq^) shows that the fluorescence associated with the target quantity (F_0_) can be calculated from F_c_, E_amc_ and Cq with the equation F_0_ = F_C_/E_amc_^Cq^. These efficiency-corrected F_0_ values can subsequently be used to calculate the relative expression (RelExp) of the target of interest (F_0,toi_) with respect to a reference gene (F_0,ref_) with RelExp = F_0,toi_/F_0,ref_. Note that it is generally recommended to use more than one reference gene. F_0,ref_ should therefore be read as the geometric mean of the expression of the reference genes [7,45,46,47].

Subsequently, the fold difference (FoldDiff) in the relative expression of the gene of interest as a consequence of a treatment, intervention or disease compared to a control or healthy condition or sample can be calculated as FoldDiff = RelExp_treated_/RelExp_control_. Note that the results of these calculations are only valid when the general assumption holds that the observed fluorescence for each amplicon copy is the same for every target. For probe assays this assumption holds true because each amplicon anneals to only one probe molecule. Even the cumulative nature of the fluorescence generated using a hydrolysis probe assay does not hamper these calculations [18]. But for DNA-binding dye assays, this assumption does not hold true because the fluorescence yield is different for amplicons of different length and, to a lesser degree, their sequence composition. To minimize such a length bias, it is recommended to stick to the same, or at least very similar, amplicon lengths for different targets. However, to eliminate this problem completely, F_0_ should be converted into N_0_, the number of copies of the target at the start of the PCR.

## 3. From F_0_ to N_copy_

As said above, the outcome of a qPCR reaction should not be reported as a Cq, a fluorescence value (F_0_) or dimensionless ratio value but preferably as the number of target copies in each reaction at the start of the PCR. We will refer to this value as N_copy_. To achieve this holy grail of qPCR, several methods have been proposed and used. For the interpretation of N_copy_, either in the number of RNA transcripts, number of pathogens or number of DNA copies in the genome, one should realize that N_copy_ is the number of target copies in the respective reaction. To obtain an insight into the number of the target of interest in the original biological sample and its biological relevance, the researcher has to convert N_copy_ into the number of molecules of the target of interest present in the original biological sample, taking into account the design of the experiment, sample collection, pre-PCR steps, assay and data analysis.

Note that the following text assumes that the qPCR assay has been validated and that the amplification of the primer-dimer or other artefacts has been checked. Reactions showing artifacts should be omitted from further analysis. Moreover, the appropriate positive and negative control samples should be included to allow quality control per run and reaction [4]. To enable the check for amplification of artefacts, it is recommended to develop the assay with DNA-binding dye monitoring of the amplification, which allows easy and sensitive detection of amplified artefacts using a melting curve analysis [48,49].

The methods described in Section 3.1 and Section 3.2 require a standard sample with a known number of target copies. These standards have to be carefully prepared to ensure the precision of the reported quantification results. The exact number of copies can be checked with a method such as digital PCR [50,51,52,53]. To ensure that this number remains constant over longer storage periods, these standards should be aliquoted to avoid freeze–thaw effects, stored in appropriate conditions and regularly checked [4,54].

### 3.1. N_copy_ Using a Calibration Curve

The most commonly used method for this so-called absolute quantification is preparing a dilution series, starting with a stock sample with a known number of target copies and using the observed Cq values to construct a calibration curve by plotting the Cq values of each reaction of the diluted calibration standard against the logarithm of the number of targets in the respective reaction. The data points of the calibration curve will be on a straight line, and Cq values of unknown samples can then be converted into the number of targets (N_copy_) in a given reaction in the same run by interpolation via this line [19]. However, this approach has the same drawbacks as the standard curve method (see Section 2.3.3.). First of all, the exact number of targets in the stock sample used to prepare the calibration curve has to be known. This caveat can be avoided by assigning a control sample and expressing the number of target copies in the unknown samples as a fraction of the number in this control.

Secondly, the actual dilution of calibration curve samples prepared from the stock sample should be the same as the intended dilution that is used in plotting the values on the x-axis. As we have shown by reanalyzing the large dataset that was used in the 2013 paper comparing the amplification curve analysis methods [21], this is not easy to accomplish. For each of the 69 genes in this large dataset, a calibration curve with five 10-fold dilution steps and three replicates per dilution was prepared using an oligo for each intended target and a robot to pipette the reactions [55]. However, the actual dilution steps for the different targets were found to range between 8- and 9.9-fold, with none of the calibration curves reaching the intended dilution of 10-fold per step [15]. Moreover, when all PCR efficiencies were determined from the amplification curves of calibration samples and unknown patient samples, the PCR efficiencies of the unknown samples differed from those of the calibration curve samples, which were prepared with only an oligo for the intended target and thus lacked the complexity of biological sample present in the patient samples [48]. Whereas a dilution error may lead to erroneous numbers on the concentration axis of the calibration curve [15], a difference in PCR efficiency between the calibration and unknown samples completely invalidates this absolute quantification approach. This is apart from the practical issues that calibration curves, like standard curves (see Section 2.3.3), have to be included in each PCR run and that they have to cover the entire, by definition unknown, range of N_copy_ possibly found in the unknown samples. An additional drawback is that Cq values outside the range of the calibration curve cannot be converted into N_copy_ because extrapolation is not allowed. This would require a costly repetition of the experiment with a wider range of dilutions of the samples used in the calibration curve.

### 3.2. N_copy_ Using Single Standard Calibration

A recently proposed, more precise and more economic approach is the single standard calibration method [15,56]. In this single standard method, replicate reactions of a standard sample with an exactly determined number of copies (preferably between 1000 and 5000 copies) are included in the PCR run without any further dilution.

When the same quantification threshold is set for the single standard reactions and the unknown reactions within a run and when, per assay or per biological sample, the mean PCR efficiency is determined from the amplification curves, the F_0_ found for the single standard samples can be used to transform the F_0_ of the unknown samples into an efficiency-corrected absolute number of target copies. This can even be achieved when the PCR efficiency of the target differs between reactions of standard and unknown samples. The underlying idea of this approach is that F_q_, the quantification threshold used in all calculations of F_0_, directly reflects the number of standard target copies (N_q_) reached at the threshold by the PCR of the standard samples as well as the PCR of the unknown samples. Because N_copy_ of the standard is known, this N_q_ can be calculated (N_q,std_ = N_copy,std_ × E_amc,std_^Cq,std^). With this N_q_ the N_copy_ of each unknown sample can be calculated as N_copy,unk_ = N_q,std_/E_amc,unk_^Cq,unk^. Note that this approach even allows the target in the unknown samples to differ from the target measured in the standard, thus obviating the need for a standard with an exactly determined N_copy_ for every target. For probe assays, F_q_ and thus N_q_ are the same for all targets, whereas for DNA-binding dye assays, this will only be true when the amplicon lengths are the same. The precision of this single standard calibration approach depends on the number of replicate reactions of the standard sample included in the run. To reach 99% precision only six such replicates have to be included, being far more economical than the large number of reactions, at least 15, needed for a calibration curve [15].

As mentioned in Section 2.3.3, the presence of PCR inhibitors in specific samples in clinical or veterinary settings will affect the PCR efficiency determined from the amplification curves [25]. In such a case, the best results will be obtained when replicate reactions per sample are used, resulting in a mean E_amc_ per specimen, rather than per target, in the calculations of the single standard calibration [15].

### 3.3. N_copy_ Using a Rule of Thumb

In an earlier attempt to estimate N_copy_, we published what we called a rule of thumb. This rule states that a reaction with an N_copy_ of 10 would result in a Cq value of 35 [57]. This rule was based on the reasoning that with a commonly used primer amount of 2.5 pmol in a 10 µL reaction and a PCR efficiency of 1.9, the number of amplicons would reach 1% of the remaining number of primer molecules after 35 cycles. At that point in the PCR, amplicon–amplicon hybridization would hamper primer annealing to the amplicon, and the reaction would go into the transition phase [11,58]. With this reasoning the kinetic equation of PCR can be written as N_q_ = 10E^35^. For a sample with unknown N_copy_, which reaches the threshold at Cq, this kinetic equation reads N_q_ = N_copy_E^Cq^. Provided the same quantification threshold is set and the same primer amount is used, N_q_ is the same in both equations. Combining both above given equations allows the calculation of N_copy_ with N_copy_ = 10E^(35-Cq)^. Note that although there is a PCR efficiency (E) in this calculation, efficiency differences are ignored. Therefore, this rule of thumb is nothing more than a quick way to estimate N_copy_ when only a Cq is given. For example, when a paper reports that Cq for a reaction is 32, then, assuming a 100% efficient PCR, N_copy_ can be estimated to be N_copy_ = 10 × E^(35-Cq)^ = 10 × 2^(35−32)^ = 10 × 8 = 80.

### 3.4. N_copy_ Using the Limiting Component Approach

Both of the above-described calibration approaches (Section 3.1 and Section 3.2) require a standard with a known number of copies of the target and thus present the user with a kind of chicken-and-egg problem: how can I determine N_copy_ in an unknown sample when I require the N_copy_ of a standard? To escape from this circular reasoning, we went back to the basics of qPCR and the characteristics of the amplification curve and extended the reasoning on which the rule of thumb (Section 3.3) for estimating N_copy_ is based. Although in each biochemical assay one aims to supply all reaction components in excess, this is often not possible due to issues with solubility and/or effects on reaction kinetics. Therefore, unavoidably the amount of one of the reaction components will become limiting, and when that happens the exponential phase of a PCR ends. What does this really mean, and does the occurrence of this unavoidable limitation hold the key to determining N_copy_ in a reaction of a given sample?

#### 3.4.1. Principle of the Limiting Component Approach

The exponential phase of a PCR is defined as the phase that shows an exponential amplification with a constant PCR efficiency. When the PCR efficiency, defined as the fold-increase per cycle, starts to decrease, the reaction enters the transition phase. Specifically, the end of the exponential phase is reached when the fold-increase in the fluorescence per cycle starts to decrease. This point in the run can mathematically be identified as the maximum in the second derivative, the so-called SDM, of the observed fluorescence data in the amplification curve [19,20,59].

In chemical terms, at the end of the exponential phase, the PCR efficiency starts to decrease because one of the reaction components becomes limiting. This can be either the primers or dNTPs required for the synthesis of amplicons or the DNA-binding dye or probes used to monitor the fluorescence associated with the number of generated amplicons. In Section 3.4.3, we will discuss how each of the different components plays a role in PCR and how the initially large but eventually limited numbers of molecules of these components affect the amplification and/or the observed fluorescence. To illustrate these effects, we prepared an Excel file (File S1) in which we simulated a qPCR reaction with standard conditions [4] and allow the user to adapt the reaction conditions to study the effects of altering the amount of each of the reaction components. In this simulation, the end of the exponential phase is assumed to occur when the PCR efficiency decreases by 0.01 from its initial value. In these simulations, the number of amplicons that can be generated before one of the reaction components becomes limiting is dubbed N_ampli_. With the kinetic equation of PCR, it can be seen that N_ampli_ = N_copy_E^SDM^. The inverse of this equation allows the calculation of the expected SDM for a given N_copy_: expected SDM = (log(N_ampli_) − log(N_copy_))/Log(E). The simulation per reaction component shows that this expected SDM is within the cycle in which the observed PCR efficiency has decreased by at least 0.01. In Section 3.4.3, the limiting effects of primers, dNTPs and fluorescent dyes and probes are individually described and discussed. These findings are then taken together and combined into a protocol to determine the N_copy_ in an individual reaction. Note that this approach requires that the assay development and validation procedures have already been performed, that the amplification of artefacts is excluded and that the appropriate positive and negative control reaction are included per run [4].

The current authors, due to their retirement, lack the laboratory facilities to validate the proposed protocol and thus cannot carry out the required validation experiments. They happily leave the validation of this limited component approach to others in the qPCR field.

#### 3.4.2. Determining the SDM of an Amplification Curve

In order to use the outlined limiting component approach to determine the N_copy_ of a given reaction, it is required that a valid SDM is determined from each amplification curve. Such an SDM is commonly calculated mathematically by fitting a parabolic [19], sigmoidal [43,59] or logistic [40] function to the (derivatives of the) observed fluorescence values of the amplification curve plotted on a linear scale. However, these non-linear models do not correctly describe the amplification curve or its second derivative. Specifically, the sigmoidal curve is strongly biased by the fit to the ground phase (all values close to 0) and the plateau phase (all values at F_max_), largely ignoring the limited number of cycles in the exponential phase, where an accurate fit is required to obtain a valid SDM [60]. Alternatively, the SDM can be graphically defined as the fractional cycle at which the curve of the third derivative of the amplification curve crosses zero, and thus the SDM of a given reaction can be determined by an algorithm that searches for this zero crossing.

#### 3.4.3. Components Limiting Amplicon Amplification

Besides the Taq-polymerase [61], the two other reaction components that are directly involved in the generation of new copies of the amplicon are the primers and the nucleotide molecules (dNTPs). Their free concentrations decrease in every cycle, but their effect on the observed amplification differs. Note that for the use of the calculations in this paragraph, the concentrations of the reaction components have to be converted into their number of molecules in the reaction (see also File S1).

- Primers

**Figure 3:**
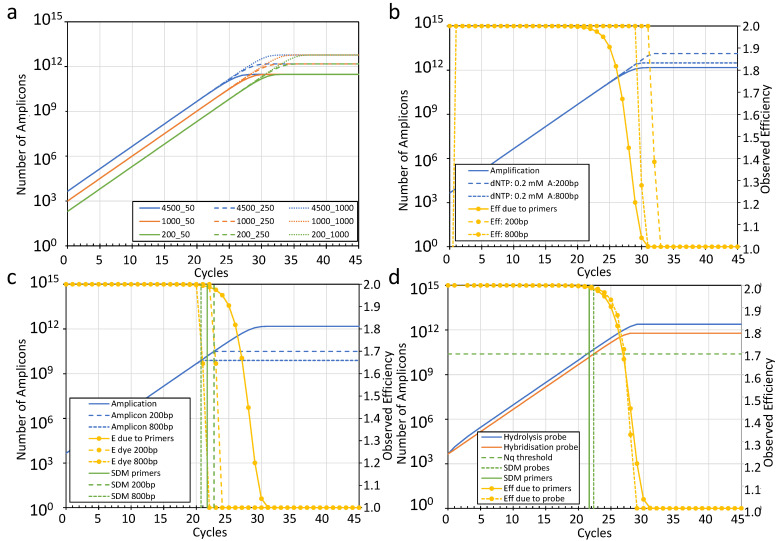
Limiting reaction components in PCR. The panels show amplification curves, the SDM and observed efficiency for different simulated scenarios. Except for the variations per panel, the reaction components are present in the standard concentration of the reaction mix (N_copy_: 4500, reaction volume: 10 μL, primers: 250 nM, dye (SYBR Green I): 98 nM, dNTP: 0.2 mM, probes: 400 nM, amplicon length 200 bp, primer length 20 bp.). (**a**) Amplification curves for different numbers of targets at the start of the reaction (N_copy_: 4500, 1000 and 200) in combination with different primer concentrations (50, 250 and 1000 nM). (**b**,**c**). Summary of the outcome of a PCR simulation when different amplicon lengths (200 and 800 bp) in relation to the limit on PCR due to dye (**b**) and to dNTP (**c**) are evaluated. (**d**) Summary of the outcome of a PCR simulation evaluating the same concentration of hydrolysis and hybridization probes. Note that although the amplification curves for the hydrolysis and hybridization probes differ, the other parameters behave the same.

The primer sequences, their composition, the local conformation at and around, their binding site in the target DNA, the composition of the reaction buffer and the annealing temperature determine the binding of the primers to the target sequence. Chemically, this annealing is a hybridization reaction that can be described with KH = [A] × [P]/[H], with KH being the hybridization constant and [A] the concentration of the amplicon, [P] of the primers and [H] of the hybrids [62]. The inverse of this equation allows the calculation of the hybrid concentration per cycle ([H] = [A] × [P]/KH). The observed in-cycle PCR efficiency (E) is calculated as the fold increase of [A] between consecutive cycles. Because of the enormous excess of primers in the reaction, KH is equal to the initial concentration of primers. Using these parameters to simulate the reaction shows that the PCR efficiency is decreased by 0.01 from its initial value, and thus by definition the SDM is reached when the [A]/[P] ratio reaches 0.01 (Figure 3a). The simulation thus shows that when the reaction reaches its SDM, the number of amplicon molecules in the reaction, dubbed N_ampli_, reaches 0.01 times the initial number of primer molecules, dubbed N_primer_, in the reaction: N_ampli_ = 0.01 × N_primer_. With the SDM and PCR efficiency determined from the amplification curve, N_copy_ can thus be calculated as N_copy_ = N_ampli_/E^SDM^. As mentioned, for the implementation of this approach, the amplification of primer-dimers or other artefacts has to be excluded in the assay [48].

- dNTPs

To evaluate whether there are conditions in which the dNTPs become limiting, a similar simulation was performed. In a standard PCR mix, 0.2 mM of each of the nucleotides were used. This number of dNTP molecules at the start of the PCR is dubbed N_dNTP_. If we assume for the simulation that all four nucleotides are equally present in the target sequence, the PCR uses two times the length of the amplicon minus the sum of the lengths of both primers in dNTP molecules per synthesized amplicon, here dubbed A_dNTP_. As long as there are sufficient dNTP molecules in the reaction mixture at the start of the elongation phase, complete amplicon molecules can be generated. However, when the number of available dNTP molecules is no longer sufficient to generate complete amplicon molecules, the exponential amplification will fail because both the forward and reverse primer target sequences need to be completely present in the synthesized amplicon for the next round of amplification. Provided all other reaction components are present in excess, the dNTPs become limiting when the number of amplicons (N_ampli_) reaches N_dNTP_/A_dNTP_. In case of an amplicon of 200 bp and primers of both 20 bp, A_dNTP_ is 360 pb, and the SDM will occur when N_ampli_ reaches (1/360=) 0.00278 times the initial N_dNTP_ in the reaction mixture. The length of the amplicon thus determines whether and when the dNTP concentration will become the limiting component of the PCR. With the standard primer concentration of 250 nM, the dNTPs will become limiting about 10 cycles after the SDM due to the primers. Only when a thousand-fold lower dNTP concentration is used does the SDM due to limiting dNTPs come close to the SDM due to limiting primers. Note that the observed PCR efficiency abruptly decreases from its initial value down to 1 in only a few cycles when the dNTPs are the limiting component (Figure 3b).

#### 3.4.4. Components Limiting Amplicon Detection

Because the presence of amplicons cannot be measured directly, indirect detection methods are used. These methods use DNA-binding dyes, which are, in general, not sequence-specific and become fluorescent upon binding to double-stranded DNA. Alternatively, there are the sequence-specific hybridization and hydrolysis probes, which bind to the single-strand denatured amplicon and become fluorescent before, or during, the elongation phase of the reaction.

- DNA-binding dyes

Many different DNA-binding dyes were developed, which all have in common that they become fluorescent when bound to double-stranded DNA (dsDNA). We have simulated the limiting reaction conditions for a dye that binds to the minor groove of the DNA helix, which is found on average at every 10.4 bp in a dsDNA helix [63,64,65]. For the sake of simplicity, we assume that amplicons adopt this dsDNA helix configuration, and as a result short amplicons bind less dye than long amplicons. The number of dye molecules that can bind to an amplicon is dubbed A_dye_ and can be calculated as the integer number of dye molecules that can bind to the amplicon: A_dye_ = int(Amplicon Length/10.4). Due to the excess of the dye at the start of the PCR, all dsDNA is fully occupied by dye in the early cycles. The number of dye molecules at the start of the PCR is dubbed N_dye_ and remains constant during the PCR. In a certain cycle during the PCR, the number of amplicons has increased to a level at which the number of available dye molecules will no longer be sufficient to completely stain all amplicons. As a consequence, the observed fluorescence in that cycle increases less than expected for the number of amplicons present in the reaction mixture. Thus, the observed in-cycle efficiency, calculated from the fluorescence between subsequent cycles, decreases, and the reaction reaches its SDM. Thus, N_ampli_ is equal to the initial number of dye molecules divided by the number of dye molecules needed to completely stain one amplicon: N_ampli_ = N_dye_/A_dye_. As observed in the simulations for the DNA-binding dye monitored PCR, the observed efficiency decreases abruptly in a few cycles.

Note that in the implementation of this approach for non-sequence-specific DNA-binding dyes, the fluorescence increase should be solely due to amplification of the intended target: artefact amplification has to be checked, and if present reactions should be excluded from the analysis. It is also notable that fluorescence due to dye binding to contaminating nucleic acids in double strand conformation, such as genomic DNA, is removed by the baseline correction.

In many commercially available qPCR kits, SYBR Green I (SGI) is used as the monitoring dye. In an initial paper describing the use of SGI in qPCR, it was stated that 98 nM SGI is the optimal working concentration for qPCR reaction mixtures [2,65,66]. In our simulations we have used this value. However, it should be noted at this point that because of insufficient information provided by the supplier/manufacturer, we cannot assume this value to be valid for all qPCR mixes. To determine the actual SGI concentration, the absorbance of its solution at 494 nm can be determined [16,65]. Alternatively, to obtain the correct dye concentration value, a pilot experiment can be performed in which samples are included with a known N_copy_. With reverse engineering of the equations, the actual dye concentration can then be determined.

Within this limitation, our simulation shows that a DNA-binding dye will only become limiting for very long amplicons or a much higher primer concentration. With a primer concentration of 2.5 pmol in a 10 µL reaction, the DNA-binding dye becomes limiting when amplicons are longer than 800 bp. With short amplicons, less than 200 bp, the dye concentration will not be the limiting factor (Figure 3c).

- Hybridization probes

Many different types of hybridization probes are used that all aim at minimal fluorescence when being free in the reaction solution and a high-level fluorescence when hybridized to their target sequence. The fluorescence of the hybridization probe has to be measured at the end of the annealing phase, when each amplicon is hybridized with a fluorescent probe. In the synthesis phase of the PCR, the probe is displaced by the advancing polymerase and stays intact but loses its fluorescence. Consequently, the amount of probe added to the start of the reaction, dubbed N_probe_, remains constant throughout the entire PCR. When the number of amplicon molecules approaches the number of probe molecules, not all amplicons will hybridize with a probe, and not all amplicons will become fluorescent; consequently, the observed efficiency is lower than expected from the number of amplicons in the reaction, and thus the SDM has been reached. The hybridization kinetics for probes are similar to those of the primers. The simulation of the probe hybridization shows that the observed in-cycle PCR efficiency decreases by 0.01 when the amplicon number reaches 0.01 times the number of probe molecules. Therefore, at the SDM, N_ampli_, due to the hybridization probe concentration, is equal to 0.01 times the initial number of probe molecules in the reaction, N_ampli_ = 0.01 × N_probe_, provided that all other components are present in excess.

- Hydrolysis probes

Another type of probes frequently used to monitor the creation of amplicons during a PCR is the so-called hydrolysis probes. Hydrolysis probes also hybridize to the amplicon during the annealing phase of the PCR. But rather than being displaced from the target like hybridization probes, these probes are hydrolyzed by the advancing Taq polymerase. When the probe is hydrolyzed, the fluorescent dye is released and becomes fluorescent, and its fluorescence accumulates during the PCR. Hydrolysis probe fluorescence should therefore be measured after the elongation phase. Note that once a probe is hydrolyzed, it is no longer available in the next cycle, and, therefore, the number of probe molecules decreases during the PCR. Despite this difference from hybridization probes, the simulation shows that the hybridization kinetics of hydrolysis probes are similar to those of primers and hybridization probes. A hydrolysis-probe-based PCR also reaches its SDM when the number of amplicons, N_ampli_, reaches 0.01 times the number of initial probe molecules: N_ampli_ = 0.01 × N_probe_. Because of the accumulative nature of the hydrolysis probe fluorescence, the exponential phase of the amplification curve of a hydrolysis probe assay is one cycle earlier than that of a hybridization probe assay applied to the same sample. The quantification threshold approach would thus give a Cq value that is one cycle lower [18]. However, the SDM of both assays is the same when both probes are initially present at the same concentration (Figure 3d).

#### 3.4.5. Calculating Ncopy Using the Limiting Components Approach

**Figure 4:**
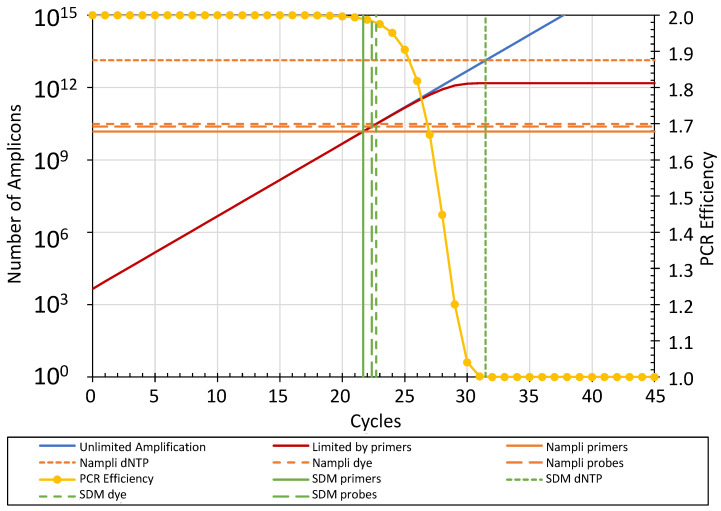
Determining the limiting reaction component in PCR. The figure shows the amplification curve limited by the primer concentration and the SDM and N_ampli_ values resulting from the other reaction components and variables for the standard reaction composition: N_copy_: 4500, reaction volume: 10 μL, primers: 250 nM, dye (SYBR Green I): 98 nM, dNTP: 0.2 mM, probes: 400 nM, amplicon length 200 bp, primer length 20 bp.

The above description of the different reaction components shows that for a specific standardized PCR design, the number of amplicons that can be generated (N_ampli_) in a reaction before the PCR efficiency drops by 0.01 can be calculated from the chosen amplicon length, primer lengths and the concentrations of each of the three reaction components: the primers, the dNTP and either the dye or the probe. These three calculations will result in three different N_ampli_ values (Figure 4). The lowest of these N_ampli_ values identifies the rate-limiting reaction component, and its value determines the number of amplicons at which the reaction in the run will reach its SDM. Note that the actual SDM value depends on the unknown N_copy_ in the reaction. With the observed SDM and the PCR efficiency (E_amc_), both determined, of course, from the amplification curve of the respective reaction, the number of target molecules at the start of the PCR can now be calculated as N_copy_ = N_ampli_/E_amc_^SDM^. As described above, the least variation in N_copy_ will be reached when the E_amc_ in this equation is determined as the mean E_amc_ of all reactions with the same target [21]. Because the PCR efficiency, E_amc_, used in the calculation is determined from replicate amplification curves, this method is not affected by PCR inhibitors present in the individual samples.

#### 3.4.6. Relative Expression and Normalization Using N_copy_

As described in Section 2.4.4, in experimental research, the expression of a gene of interest is generally reported relative to the expression of a set of validated reference genes [4,46]. To this end the expression of the gene of interest is divided by the average expression of the reference genes, calculated as the geometric mean of the expression of the reference genes per sample. This procedure can also be applied to N_copy_ values. However, this regretfully leads to loss of information about the absolute gene expression level because the result of this calculation is a fold difference compared to the reference genes. It is a pity that we thus lose the informative clarity of the absolute N_copy_ values that we have just determined. However, with a simple extra step, the absolute number of target copies can be retained. By definition, the average expression of the reference genes is the same for all samples in the experiment. Therefore, the above relative results will again reflect the absolute number of target copies when they are multiplied by the geometric mean of the N_copy_ values of all reference genes in all samples in the experiment.

## 4. Discussion and General Conclusions

Taken together, as discussed and illustrated above, one can calculate the target quantity in the reaction at the start of the PCR (N_copy_) from the known amount of each of the reaction components in a given PCR, its PCR efficiency (E_amc_) and SDM determined from the amplification curve. This N_copy_ is assay-, machine- and laboratory-independent and allows direct comparison of qPCR data between different laboratories and provides a very intuitive and easy-to-interpret absolute quantitative result. This is a goal that, despite the efforts to standardize the reporting of all steps of a qPCR experiment [4,8], was never reached. In our view, reporting N_copy_ rather than Cq, solves most of the reproducibility issues of qPCR [11].

A few considerations have to be addressed before implementing the discussed limiting component approach to determining N_copy_. Firstly, one should take into account that the SDM is defined as the fractional cycle at which the PCR efficiency has decreased with 0.01 from its initial value. However, in the calculation of N_copy_, the original efficiency value, which by now deviates by 0.01 from the observed value, is used. The simulation of the role of primer concentrations shows that this discrepancy leads to a negative bias of 0.005 in N_copy_. Compared to other errors in qPCR, this bias can be considered to be negligible.

Secondly, the simulations of dye and dNTP limits revealed that both components show an abrupt decrease in the observed PCR efficiency within one cycle. Because, contrary to the situation of the primer hybridization, we are not aware of a model to describe the efficiency loss within this cycle, it was not possible to calculate an exact SDM from the simulated amplification curve. However, the fact that the expected SDM always falls within this cycle shows that the determined limits and thus N_ampli_ for those reaction components are most probably correct.

Finally, we are aware that wide implementation of this limiting component approach requires that the qPCR users know the exact compositions of their reaction mixes. This requirement will increase the awareness of the need for standardization of qPCR [4] and hopefully convince manufacturers of qPCR kits to disclose the compositions of the master mixes. On the other hand, the required calculation of N_ampli_ per reaction component will give insight into the rate-limiting factors in the PCR assay and may help with optimization of qPCR.

## Data Availability

No new data were created or analyzed in this study. Data sharing is not applicable to this article.

## Supplementary Materials

The following supporting information can be downloaded at: https://www.mdpi.com/article/10.3390/ijms262411885/s1.

## Abbreviations

PCR	Polymerase Chain Reaction
qPCR	quantitative Polymerase Chain Reaction
toi	target of interest
ref	reference gene
unk	unknown sample
N_0_	number of copies of the target at the start of the PCR
N_C_	number of copies of the target after C cycles
C	number of cycles
E	efficiency of the amplification reaction
F_0_	fluorescence of the target molecules at the start of the PCR
F_C_	fluorescence of the copies of the target after C cycles
F_q_	quantification threshold
Cq	quantification cycle
ΔCq	difference between the Cq values of toi and ref
ΔΔCq	difference between the ΔCq values of control and treated groups
Eamc	amplification-curve-derived PCR efficiency
Estc	standard-curve-derived PCR efficiency
SDM	maximum of the second derivative of the amplification curve
RelExp	relative expression
FoldDiff	fold difference
Ncopy	number of target copies at the start of the PCR
Nampli	maximum number of amplicons due to a limiting reaction component
KH	hybridization constant
[A]	concentration of the amplicon
[P]	concentration of the primers
[H]	concentration of the hybrids
Nprimer	number of primer molecules
NdNTP	number of dNTP molecules
AdNTP	number of dNTP molecules needed to synthesize one amplicon
Ndye	number of DNA-binding dye molecules
Adye	number of dye molecules needed to stain one amplicon
SGI	SYBR Green I
Nprobe	number of probe molecules
